# POTENTIAL PROTECTIVE EFFECTS OF INSTRUMENTAL ACTIVITIES OF DAILY LIVING ON ALZHEIMER’S DISEASE CHARACTERISTICS

**DOI:** 10.1093/geroni/igad104.2585

**Published:** 2023-12-21

**Authors:** Rachel McCray, Daisha Oruru, Clarence Locklear, Omonigho Bubu, Arlener Turner

**Affiliations:** New York University, New York City, New York, United States; University of Miami, Miller School of Medicine, Miami, Florida, United States; University of Miami, Miller School of Medicine, Miami, Florida, United States; New York University, Grossman School of Medicine, New York City, New York, United States; University of Miami, Miller School of Medicine, Miami, Florida, United States

## Abstract

Previous research has shown the significance of self-efficacy in improving cognitive trajectory. Additionally, it has been demonstrated that impaired cognitive functioning is a predictor for low functionality in both basic and instrumental activities of daily living (iADLs). However, little research has been done on potential protective effects of iADL functionality on markers of Alzheimer’s disease. To examine if iADL functionality (possibly through self-efficacy) has a protective effect on Alzheimer’s disease characteristics in older adults, we assessed the relationship between functionality and hippocampal atrophy. This study utilized data from the National Alzheimer’s Coordinating Center Uniform Dataset (NACC UDS) covering September 2005 to February 2021. Participants included in the analyses (N=2517, 65.7% women, 11.9% Black/African American, mean age 68.7, SD=9.15) were all cognitively normal. The analyses use baseline Functional Activities Questionnaire (FAQ) assessment, measures of independent living, and required assistance, in addition to presence or absence of Hippocampal Atrophy (measured ~5 years later) in two 2x2 contingency tables. While no relationship between baseline FAQ and later hippocampal atrophy emerged, a relationship between independent living status at baseline and hippocampal atrophy was demonstrated (X2{2,446}=11.38, p=0.003) with higher independence in iADL tasks associated with less atrophy. Though further research is needed, these preliminary results suggest living independently and having agency over iADL tasks may confer some neurocognitive protection and indicate the possibility of less hippocampal atrophy over time in individuals with higher levels of independence. Future studies should also explore the potential positive influence of iADLs on other characteristics of Alzheimer’s disease like cognition.

